# SARS-CoV-2 Infections in a High-Risk Migratory Population Arriving to a Migrant House along the US-Mexico Border

**DOI:** 10.3390/tropicalmed7100262

**Published:** 2022-09-24

**Authors:** Nadia A. Fernández-Santos, Gabriel L. Hamer, Edith G. Garrido-Lozada, Mario A. Rodríguez-Pérez

**Affiliations:** 1 Instituto Politécnico Nacional, Centro de Biotecnología Genómica, Reynosa 88710, Mexico; 2 Department of Entomology, Texas A&M University, College Station, TX 77843, USA; 3 Daughters of Charity of Saint Vincent de Paul (La Casa del Migrante), Reynosa 88520, Mexico

**Keywords:** COVID-19, SARS-CoV-2, México, migrants, infectious diseases, prevention

## Abstract

Few reports exist on the COVID-19 epidemiology of migrant populations. We tested 370 migratory individuals from ten countries arriving at a migrant house along the US–Mexico border based on a rapid assay detecting SARS-CoV-2 antigen. Fifty-six were positive, for a prevalence of 15.1% (95%–CIs of 11.8–19.2%). Only 21 positive persons presented signs or symptoms associated with the infection (95%–CIs = 25–49%). Most (51.7%) positive migrants arrived in the previous two days before being tested, indicating that the virus infection was acquired during their transit. Out of the total of 56 positive individuals, 37.5% were from El Salvador, 33.9% from Honduras, and 21.4% from Guatemala. This study suggests that vulnerable populations traveling from countries in Latin America and seeking residence in the US are high-risk individuals for exposure to SARS-CoV-2. The rapid antigen COVID-19 testing on arrival at the migrant house, and subsequent 10-day quarantine, was a critical step to help minimize further transmission. Therefore, the present study demonstrates that public health services provided to migratory and vulnerable populations are necessary for pandemic control.

## 1. Introduction

The COVID-19 pandemic declared by the World Health Organization (WHO) on March 11, 2020, put the entire world in an unprecedented health crisis, which has led to a state of persistent uncertainty [1]. The groups most seriously affected by this health crisis are migrants and refugees, due to the increase in inequalities generated by the pandemic [2,3]. COVID-19 has emerged in a world closely connected with local and international population movements and with a greater number of people who move for work, education, family, tourism, and survival [4]. Migratory movements are dynamic and result from fundamental demographic, social, cultural, and economic phenomena that shape the local context where the pandemic, residents, and migrants co-exist [5]. Furthermore, geographic and geopolitical position, relative level of wealth, and international connections make some countries attractive destinations for migrant workers, international students, asylum seekers, and refugees [6]. The high number of migrants in these attractive countries underscores the specific need to include migrants in response and recovery efforts from the effects of COVID-19.

Organized caravans of persons from Central American countries traveling from southern to northern Mexico [7] results in many migrants being barred for months, and even years, at the borders of the different countries of origin and destination [8,9]. In this context of temporary housing, often in high densities, the transmission of SARS-CoV-2, the agent of COVID-19, can be high among resident and migrant populations. The COVID-19 problem is further exacerbated in migrant shelters, given the unsanitary conditions that prohibit basic preventive health measures. Migrants do not practice social distancing; there is absent or low coverage of face masks, no proper washing or regular hand washing, and little or no use of disinfectants, so the risk of exposure to SARS-CoV-2 is high in these populations [10,11]. In Mexico, as well as in other countries with a migrant population, support work for migrants is challenging; migrants often sleep in crowded closed dormitories with poor or no ventilation and sanitation, work illegally without decent conditions, or experience severe mobility restrictions, under different levels of scrutiny and/or facing public suspicion, even opprobrium, in a foreign country [10,12,13]. The Mexican Immigration Policy Unit (Registration and Identity of Persons) reported the delivery of 66,685 visitor cards for humanitarian reasons during 2020 at 2022 (March) [14]. Multiple entries are given through Tapachula city in Chiapas to migrants traveling mostly in caravans. From 2020 to March 2022, US Customs and Border Protection detected 3,488,674 undocumented immigrants on the border with Mexico [15]. In 2021, Mexico became the third country in the world to receive the largest number of new asylum applications [16]. People from various countries, including Haiti, countries in Central America, Venezuela, Cuba, the African continent, and elsewhere, submitted 562,549 asylum applications [16].

Those migrants, asylum seekers, and US deportees converge and concentrate mostly in 90 shelters run commonly by local government, religious, and non-governmental organizations (NGOs) located along nine northern Mexico cities: Ciudad Juarez, Mexicali, Reynosa, Nuevo Laredo, Rio Bravo, Matamoros, Nogales, Piedras Negras, and Tijuana [17], which are reference point areas of convergence and transit of the different demographic groups. Annually, Mexicans and ca. 50 other nationalities looking for temporary residence seek these destinations with the ultimate goal of reaching different cities in the US. Additionally, shelters in the Mexican territory are also receptor centers of US-expelled people (deportees) coming by land mainly from the nine processing offices of the US Southwest Customs and Border Protection Office of the Department of the Homeland Security [15]. These non-permanent people, which number in the thousands every year, are considered among the most vulnerable individuals in the social fabric of the border population, and this long interchange of people on both sides of the US-Mexico border creates a high risk for SARS-CoV-2 transmission.

The metropolitan city of Reynosa is the location of “The Migrant House” (TMH), which is run by the Daughters of Charity of Saint Vincent de Paul. This house offers a dignified and safe place to migrants who arrive in Reynosa, providing them with free basic services during their stay, such as food, clothing, personal hygiene items, telephone calls, medical attention, psychological attention, paperwork, and administration of personal documents. Within these facilities, a “Médecins Sans Frontières” (MSF) team provides medical and psychological consultations to this migrant population, but it also does so along its migratory route, prioritizing assistance to the most vulnerable groups. Likewise, other NGOs work for and with the migrants of this house. Due to the COVID-19 pandemic, they were forced to remain, indeterminately, in Reynosa. Migrants must cohabit in large groups of up to 20 people per dwelling; likewise, the temporary jobs to which they have access are of high risk for them and the local population. For example, cleaning the windshields of cars at traffic lights inso Reynosa is one form of income. This is in addition to the high-risk situation for COVID-19, since the Reynosa municipality officially presents the highest number of COVID-19 cases in Tamaulipas. As of 9 August 2022, the official figures in Reynosa were 24,388 confirmed cases of COVID-19 with 1628 deaths, which represents 14% and 20.4% of the total confirmed cases and deaths, respectively, for Tamaulipas [18]. During 2019–2022, TMH in Reynosa sheltered over 11,000 migrants; out of these, 10,548 were Mexicans, and 883 were from other countries. In total, 88% percent of the migrant population was male, and the remaining 12% was female and minors.

## 2. Materials and Methods

The study was carried out at “The Migrant House” (26°05′49.8″ N; 98°17′12.0″ W) in Reynosa, México. This was a cross-sectional study conducted from August to November, 2021 [19]. The inclusion criteria were all migrants, excluding newborns less than one year old. Eligible people arriving to TMH were continuously recruited to participate in the study and sampled until test supplies were exhausted (370 total migrants). A rapid lateral flow assay detecting SARS-CoV-2 antigen (PANBIO Covid-19 Ag rapid test; Abbott Laboratories de Mexico, S.A. de C.V. No. 41FK10) with nasopharyngeal swabs was used by health workers to test migrants. The specificity and sensitivity of this rapid test has been estimated by the manufacturer at 91.4 % (94.1 % for samples showing Ct-values = ≤33) and 99.8%, respectively (*vs*. nasopharyngeal PCR) [20]. However, the sensitivity may vary depending upon the time of testing and days from the onset of symptoms. Thus, a prevalence of 10.4% and sensitivity of 79.6% (95–CIs = 67.0–88.8%) was reported when 412 symptomatic patients of healthcare centers in Spain were tested with the PANBIO rapid test [21].

We tested migrants on arrival to TMH; if the person tested positive, they were required to isolate for a 10-day quarantine period outside the communal environment per local health authorities. This strategy allowed TMH to continue to provide essential services to migrants while minimizing SARS-CoV-2 transmission, as migrants showed active and recent infections with SARS-CoV-2. The present study was conducted during the third COVID-19 “wave” in Mexico, when the incidence rate in Tamaulipas was 1.09% (maximum incidence of 3.39% in Mexico city) [19].

Statistical analysis. The 95% exact Bayes confidence intervals (95%–CIs) surrounding the point estimate of the COVID-19 prevalence was calculated as previously reported [22].

## 3. Results

We tested 370 migrants from ten countries, including 35.9% from Honduras, 26.4% from El Salvador, 18.3% from Guatemala, and 12.9% from Mexico (plus 1.6% from TMH staff). The other countries (Nicaragua, Venezuela, Haiti, United States, Colombia, and Ecuador) were less abundant, ranging from 0.2% to 1%. Of the 370 tested, 56 were positive to the antigen test (prevalence of 15.1%; 95%–CIs of 11.8–19.2%; Table 1). The refusal rate was 0.81%, as only three migrants were reluctant to participate and were, therefore, not admitted to enter the TMH.

The percent of migrants that tested positive by nationality ranged from 0% to 50%. By country of origin, we observed 50% prevalence (1 positive of 2 tested) of the individuals from Colombia, 25% from Venezuela (1 of 4), 21.4% from El Salvador (21 of 98), 17.6% from Guatemala (12 of 68), 14.2% from Honduras (19 of 133), and 4.17% from México (2 of 48). The other countries (Nicaragua, Haiti, United States, and Ecuador) and TMH staff had no positive cases of the people tested (Table 1). Thus, the top four positive for SARS-CoV-2 were: 21 people (37.5% of total positives) from El Salvador, 19 (33.9%) from Honduras, 12 (21.4%) from Guatemala, and 2 (3.6%) from Mexico. Only one person (1.8%) tested positive from the four and two people examined from Venezuela and Colombia, respectively.

Of the migrants positive for the SARS-CoV-2 antigen test, only 21 persons presented signs or symptoms associated with the infection (95%–CIs = 25–49%); the remaining 63% of positive persons were asymptomatic. The migrants testing positive by age group were as follows: children between 0 and 20 years old, 18.4% (*n* = 163); followed by adults between 21 and 50 years old, 13.1% (*n* = 199); and elderly people between 51 and 70, 0% (*n* = 8). By gender, 135 and 235 men and female were examined, of which 25 and 31 were positive, respectively (Table 2).

**Figure 1:**
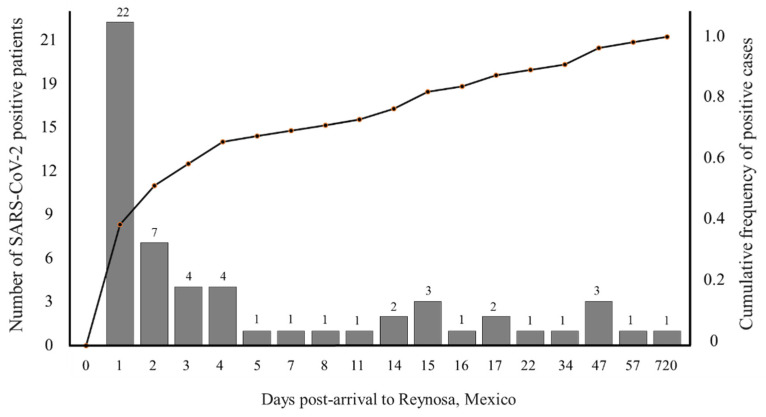
Cumulative frequency of the number of individual migrants arriving to “The Migrant House” in Reynosa, Mexico, that tested positive SARS-CoV-2 antigen at different days post-arrival.

Most (51.7%) positive migrants arrived to Reynosa within two days of being tested (Figure 1), suggesting that exposure to SARS-CoV-2 occurred during travel from their country of origin. The average amount of time for migrants to travel from southern to northern Mexico is 24 to 72 h according to the answers of 60 migrants, who responded to standardized questionnaires (unpublished data), which includes the incubation period for the delta variant, the most predominant variant of concern circulating in Mexico during this study [23,24].

Integrated health services supported by the network of TMH, MSF, and NGOs are paramount to improving access to rapid free tests in vulnerable populations [25], because testing for virus infection is critical to detecting COVID-19 cases for quarantine and contact tracing, as well as for providing medical care. Undoubtedly, migrants are a high-risk population for virus infection; it has been reported that migrants are disproportionately represented among COVID-19 cases and deaths. Risk factors for migrants include high-risk occupations, overcrowding, and barriers to healthcare, including imprecise information, languages, and limited human rights [26]. For example, in Saudi Arabia and Singapore, low-skilled migrant workers in crowded dormitories showed that 75 and 95%, respectively, of all newly confirmed cases were among migrants, and 93% of total cases were associated with migrants’ dormitories in Singapore [27]. Although migrants depend heavily upon services of lodging, food, and medical care provided by the charitable network of TMH, MSF, and NGOs in Reynosa, this network lacks sufficient resources to provide all the needed services. The current scenario for migrants in northern border cities is worrisome, as expulsions from the US are ongoing. In addition, migrants seeking asylum in the US travel from Tapachula, a southern Mexico border city, to US–Mexico border cities. Here, the needs of migrants overwhelm the capacity of local health authorities and NGOs [28]. The emergence of new SARS-CoV-2 variants of concern will likely continue to result in epidemic waves. Thus, there is an urgent need to enhance medical care for this migrant population. Further studies are needed to confirm the role of migratory populations relative to resident populations in the spread of SARS-CoV-2 and similar emerging infectious diseases.

A limitation of the current study is that we were unable to confirm exactly when and where prior exposure to SARS-CoV-2 occurred for the migrants arriving to TMH in Reynosa. While we report variation among populations of migrants coming from different countries of origin, this prevalence does not necessarily reflect infection from the country of origin, and instead could reflect variation in the risk of exposure to SARS-CoV-2 during transport or staging in different locations along the route to the US.

## 4. Conclusions

Here, we tested 370 migrants from ten countries arriving at the US–Mexico border, and 56 tested positive for the SARS-CoV-2 antigen, which triggered a quarantine protocol to limit further spread. Thus, the present study documents a screening protocol of individuals arriving at TMH to guide actions for the prevention and control of SARS-CoV-2. Diagnostic rapid free testing for migratory populations is important for providing public health services to vulnerable populations that could contribute to SARS-CoV-2 spread [23].

## Figures and Tables

**Table 1:** Percentages of migrants from 10 countries examined and tested positive for SARS-CoV-2.

Country	No. Examined	% in Relation to Total Examined	No. Positives	% in Relation to Total Positives	% of No. Positives/Examined per Country
Colombia	2	0.5	1	1.8	50.0
Venezuela	4	1.0	1	1.8	25.0
El Salvador	98	26.4	21	37.5	21.4
Guatemala	68	18.3	12	21.4	17.6
Honduras	133	35.9	19	33.9	14.2
Mexico	48	12.9	2	3.6	4.1
Mexico (TMH *)	6	1.6	0	0	0.0
Nicaragua	4	1.0	0	0	0.0
Haití	3	0.8	0	0	0.0
USA	3	0.8	0	0	0.0
Ecuador	1	0.2	0	0	0.0
Total	370	100	56	100	15.1

* TMH = The Migrant House staff.

**Table 2:** Testing results (no. of positives/no. of examined) for SARS-CoV-2 antigen in nasopharyngeal swabs of migrants arriving to “The Migrant House” in Reynosa, Mexico, according to country of origin, gender, and age group.

Country	Males	Females	0–20 Years Old	21–50 Years Old	51–70 Years Old
Honduras	7/42 (31.1)	12/91 (38.7)	10/59 (16.9)	9/72 (13)	0/2 (0.0)
El Salvador	7/30 (22.2)	14/68 (28.9)	14/48 (29.1)	7/49 (14)	0/1(0.0)
Guatemala	7/28 (20.7)	5/40 (17)	6/30 (20)	6/37(16)	0/1 (0.0)
Mexico	2/21 (15.6)	0/27(11.5)	0/17(0.0)	2/28 (7.1)	0/3 (0.0)
Venezuela	1/4 (3)	ND	ND	1/3 (33.3)	0/1 (0.0)
Haití	0/3 (2.2)	ND	ND	0/3 (0.0)	ND
Mexico (TMH *)	0/2 (1.5)	0/4 (1.7)	0/3 (0.0)	0/3 (0.0)	ND
Colombia	1/2 (1.5)	ND	ND	1/2 (50)	ND
Ecuador	0/1 (0.7)	ND	0/1(0.0)	ND	ND
Nicaragua	0/1 (0.7)	0/3(1.3)	0/2 (0.0)	0/2 (0.0)	ND
USA	0/1 (0.7)	0/2(0.9)	0/3 (0.0)	ND	ND

* TMH = The Migrant House staff. ND = no data (no individuals in this category).
